# Exploring the allelopathic autotoxicity mechanism of ginsenosides accumulation under ginseng decomposition based on integrated analysis of transcriptomics and metabolomics

**DOI:** 10.3389/fbioe.2024.1365229

**Published:** 2024-03-07

**Authors:** Rui Wang, Tingting Zhou, Yikai Wang, Jinxu Dong, Yutao Bai, Xin Huang, Changbao Chen

**Affiliations:** Jilin Ginseng Academy, Changchun University of Chinese Medicine, Changchun, Jilin, China

**Keywords:** ginsenoside, decomposition, transcriptomics, metabolomics, integrated analysis, allelopathic autotoxicity

## Abstract

Continuous cropping obstacles seriously constrained the sustainable development of the ginseng industry. The allelopathic autotoxicity of ginsenosides is the key “trigger” of continuous cropping obstacles in ginseng. During harvest, the ginseng plants could be broken and remain in the soil. The decomposition of ginseng residue in soil is one of the important release ways of ginsenosides. Therefore, the allelopathic mechanism of ginsenosides through the decomposed release pathway needs an in-depth study. To investigate this allelopathic regulation mechanism, the integrated analysis of transcriptomics and metabolomics was applied. The prototype ginsenosides in ginseng were detected converse to rare ginsenosides during decomposition. The rare ginsenosides caused more serious damage to ginseng hairy root cells and inhibited the growth of ginseng hairy roots more significantly. By high-throughput RNA sequencing gene transcriptomics study, the significantly differential expressed genes (DEGs) were obtained under prototype and rare ginsenoside interventions. These DEGs were mainly enriched in the biosynthesis of secondary metabolites and metabolic pathways, phytohormone signal transduction, and protein processing in endoplasmic reticulum pathways. Based on the functional enrichment of DEGs, the targeted metabolomics analysis based on UPLC-MS/MS determination was applied to screen endogenous differential metabolized phytohormones (DMPs). The influence of prototype and rare ginsenosides on the accumulation of endogenous phytohormones was studied. These were mainly involved in the biosynthesis of diterpenoid, zeatin, and secondary metabolites, phytohormone signal transduction, and metabolic pathways. After integrating the transcriptomics and metabolomics analysis, ginsenosides could regulate the genes in phytohormone signaling pathways to influence the accumulation of JA, ABA, and SA. The conclusion was that the prototype ginsenosides were converted into rare ginsenosides by ginseng decomposition and released into the soil, which aggravated its allelopathic autotoxicity. The allelopathic mechanism was to intervene in the response regulation of genes related to the metabolic accumulation of endogenous phytohormones in ginseng. This result provides a reference for the in-depth study of continuous cropping obstacles of ginseng.

## 1 Introduction

Ginseng (*Panax ginseng* C. A. Meyer) is a famous traditional Chinese medicine with high medicinal, nutritional, and healthcare value (Shen et al., 2023). After the cultivation of ginseng, the soil becomes unsuitable for ginseng continuous cropping. The growth and development are inhibited and weakened, which lead to serious root rot, fibrous root shedding, and even plant death. Therefore, the secondary planting rate of ginseng would be seriously reduced (Tagele et al., 2023). Continuous cropping obstacles in ginseng have seriously restricted the development of the ginseng industry. The recycling of land needs to be solved urgently. The reason for continuous cropping obstacles in ginseng is very complex. The changes in microbial communities and physicochemical properties of soil, the autotoxicity of allelochemicals, and the interactions of factors could cause continuous cropping obstacles (Liu et al., 2021; Chen et al., 2023a; Jia et al., 2023). The allelopathic autotoxicity refers to the inhibition of growth and development of the same plant species by allelochemicals. Ginsenosides are the main allelochemicals in ginseng and their autotoxicity plays an important role in ginseng continuous cropping. The decomposition of ginseng residue in the soil during harvesting is one of the release ways of allelochemicals, which could affect soil structure and ginseng growth directly or indirectly (Singh et al., 2021).

Phytohormones refer to trace organic compounds that are synthesized in the plant body, which have a significant regulatory effect on the growth and development of plants (Bogatek and Gniazdowska, 2014; Ohri et al., 2015). Common phytohormones include auxin, cytokinins (CKs), abscisic acid (ABA), jasmonates (JAs), salicylic acid (SA), gibberellins (GAs), ethylene class (ETH), and strigolactones (SLs). The physiological activities of phytohormones are related to their contents in plants. Studies on the composition and content changes of phytohormones in plants can help to find more interactions of phytohormones and explore possible mechanisms (Pan et al., 2010; Jiang and Asami, 2018).

By transcriptome sequencing, plant-specific key genes, function prediction, and metabolic pathways could be obtained. The adversity-related regulatory networks could be explored (Wan et al., 2022; Chen et al., 2023b). Metabolomics is the study of the changes of small molecule metabolites in a biological system before and after a perturbation during a specific physiological period (Ren et al., 2016), and the overall metabolic profile of the biological system could be characterized and the mechanisms could be clarified. Plant transcriptomics is the study of gene expression at the entire RNA transcript level in plants (Zanardo et al., 2019). Metabolomics provides more epigenetic data to support transcriptomics (Salek et al., 2015). The functional changes in genes are ultimately reflected at the metabolic level, and metabolites can amplify the expression of these genes at the functional level. Integrated transcriptomics and metabolomics analysis of biological systems can provide a more systematic and comprehensive understanding of the molecular functions and regulatory mechanisms (Wu et al., 2023; Zhang et al., 2023).

At present, the allelopathic autotoxicity mechanism of ginsenosides by decomposition as a release way needs an in-depth study. In this study, we simulated the natural decomposition process of ginseng in the soil. After decomposition, the ginsenoside composition of ginseng was analyzed comprehensively using UPLC-HRMS and the transformation was explored systematically. The allelopathic autotoxicity of ginsenosides was validated by the apparent morphology and apical cell viability of ginseng hairy roots. Transcriptomics sequencing was applied to screen the gene expression. Furthermore, a targeted metabolomics approach based on UPLC-MS/MS was applied to study the endogenous phytohormones metabolism in ginseng hairy roots. Finally, the analysis of transcriptomics and metabolomics were integrated to explore the allelopathic autotoxicity mechanism of ginseng decomposition deeply.

## 2 Materials and methods

### 2.1 Materials

#### 2.1.1 Reagents

Methanol and acetonitrile (HPLC grade, Tedia), formic acid and acetic acid (HPLC grade, Aladdin), n-butanol (analytical grade, Tianjin Xintong Fine Chemical Co., Ltd.), phytohormone standards and internal standards (TRP, SAG, IAA-Glc, etc. Sigma-Aldrich), and ultrapure water (Milli-Q water, Merck Millipore).

#### 2.1.2 Plant materials and decomposition model preparation

Fresh ginseng root (6 years old) was collected from the plantation of Baixi Forestry, Fusong County, Baishan City, Jilin Province. It was identified by Prof. Chen Changbao of Changchun University of Chinese Medicine as *Panax ginseng* C. A. Meyer.

Decomposition model preparation: the main root part of ginseng was cleaned, wipe-dried, cut into small pieces, and mixed with forest soil (1:20, w/w). The model samples were placed in a ventilated area with natural light and soil humidity maintained at 10%–15%. We allowed the ginseng root to decay naturally in the forest soil for 1 year and collected the soil for further testing. Each treatment was repeated five times.

### 2.2 Analysis of ginsenoside fractions

#### 2.2.1 Extraction of ginsenosides

A number of fresh ginseng main root were removed and dried at 45°C, pulverized, and passed through a 60-mesh sieve for further extraction. The soil with decomposed ginseng was mixed thoroughly, dried at 45 °C, ground, and passed through a 50-mesh sieve for further extraction.

We weighed the ginseng root sample (1.0 g) and soil sample (30 g) and added 10 times the amount of 50% methanol; they were ultrasonic extracted at 50°C two times and each time for 1 h, and we combined the extracts and concentrated them under reduced pressure to 10 mL. An equal volume of water-saturated n-butanol was added and extracted three times, and the n-butanol layers were combined and concentrated under reduced pressure till they were dry. The extract was dissolved using HPLC grade methanol to 1 mL and filtered with 0.22 μm Millipore for UPLC-HRMS analysis.

#### 2.2.2 UPLC-HRMS determination of ginsenosides

Ultra-high performance liquid chromatography-quadrupole electrostatic field orbitrap high-resolution mass spectrometer (UPLC-HRMS) (Q-Exactive, Thermo-Fisher, United States) and Thermo Scientific Syncronis C18 column (100 mm × 2.1 mm, 1.7 μm) were used.

Chromatographic conditions: mobile phase: 0.1% formic acid water (A) and acetonitrile (B); gradient elution: 0–38 min 15%–80% B, 38–40 min 80%–100% B, 40–45 min 100% B; flow rate: 300 μL/min; column temperature: 30 °C; injection volume: 5 μL.

Mass spectrometry conditions: full scan under electrospray ionization (ESI) in negative ion mode; spray voltage: 2500 V; sheath gas: 40 arb; aux gas: 15 arb; capillary temperature: 320 °C; scan range: *m/z* 150-2000.

### 2.3 Validation of allelopathic autotoxicity

#### 2.3.1 Ginseng hairy roots culture

A single cell line of ginseng tissue was applied to induce hairy root growth by Agrobacterium rhizogenes. The grown hairy roots were transferred several times to remove Agrobacterium rhizogenes. Then the ginseng hairy roots were inoculated into a solid medium (Meng et al., 2022). After the growth of ginseng hairy roots stabilized, 1 cm of the root tip was transferred to different groups of solid mediums for culture. Six biological replicates were set up in each group. For the G group, 1 mL of ginseng root aqueous extracts was added to the solid medium, and the total ginsenoside contents of ginsenosides in the solid medium were 0.01 mg/L, 0.1 mg/L, 1 mg/L, and 10 mg/L, respectively. For the DG group, an equal volume of aqueous extracts of decomposed ginseng products was added to the solid medium, and the total ginsenoside contents of ginsenosides in the solid medium were 0.01 mg/L, 0.1 mg/L, 1 mg/L, and 10 mg/L, respectively. For the C group, an equal volume of distilled water was added to the solid medium. The growth status of hairy roots was observed. Hairy root samples were collected after 30 days of culture and stored at −80 °C for transcriptome sequencing and metabolomics analysis.

#### 2.3.2 Determination of ginseng hairy roots cell viability

The ginseng hairy roots were soaked in FDA-PI double stain and incubated for 30 min without light. The excess dye was removed by rinsing the root in saline. The slices were sealed with an anti-fluorescence quenching sealing solution and placed under a laser confocal microscope with excitation and detection wavelengths at 485 nm and 530 nm, respectively, to observe the cell viability of hairy roots.

### 2.4 Transcriptomics analysis

#### 2.4.1 RNA extraction and detection

We took 100 mg of ground ginseng hairy roots and added 1 mL of CTAB solution. The sample was placed in a thermostatic mixer, mixed at 60 °C for 10 min, and centrifuged at 12,000 rpm for 5 min. The supernatant was transferred to an equal volume of chloroform, mixed well, centrifuged at 12,000 rpm for 5 min, and repeated twice. The supernatant was transferred to an equal volume of LiCl solution, mixed well, placed at −20 °C for 2 h, centrifuged at 12,000 rpm for 20 min, and discarded. Ethanol of 75% was added, mixed well, and centrifuged at 12,000 rpm for 5 min, and the supernatant was discarded. The supernatant stood at room temperature for 5 min, and 30 μL of DEPC-treated water was added, mixed well with vortex, and stored at −80 °C for measurement.

The integrity of RNA was analyzed using agarose gel electrophoresis and Agilent 2100 bioanalyzer. The purity of RNA was measured by a Nano-spectrophotometer. The concentration of RNA was quantified accurately by Qubit 2.0 Fluorometer.

#### 2.4.2 Library preparation for transcriptome sequencing

Sequencing libraries were generated using NEBNext® Ultra™ RNA Library Prep Kit of Illumina® (NEB, United States). The first strand of cDNA was synthesized in the M-MuLV reverse transcriptase system using the fragmented mRNA as a template. The second strand synthesis of cDNA was subsequently performed using DNA Polymerase I and RNase H. The purified double-stranded cDNA was end-repaired, A-tailed, and connected to the sequencing connector. Fragment selection was performed with AMPure XP beads software. The sorted products were PCR amplified and purified to obtain the final library. After the library was constructed, preliminary quantification was performed using a Qubit 2.0 Fluorometer, and then the insert size of the library was examined using an Agilent 2100 bioanalyzer. The effective concentration of the library was quantified accurately by qRT-PCR to ensure the quality of the library.

#### 2.4.3 Transcript assembly, gene annotation, and functional classification

Transcriptome assembly was performed using Trinity. Corset was used to regroup relevant transcripts into ‘gene’ clusters (https://github.com/trinityrnaseq/trinityrnaseq). DIAMOND software was used to compare the transcript sequences after redundancy with KEGG (https://www.genome.jp/kegg/), NR (http://www.ncbi.nlm.nih.gov/), Swiss-Prot (https://www.expasy.org/resources/uniprotkb-swiss-prot), GO (https://www.geneontology.org/), COG/KOG (http://ftp.ncbi.nih.gov/pub/COG/KOG/kyva), and TrEMBL (a variety of new documentation files and the creation of TrEMBL, a computer annotated supplement to Swiss-Prot) databases. HMMER software was used to compare amino acid sequences with the Pfam (http://pfam.xfam.org/) database. The annotation information of transcripts in seven major databases was obtained. Gene expression levels were calculated by RSEM. Based on the gene length, the FPKM of each gene was calculated. Analysis of variance was performed using DESeq2, with *p*-values and |log2foldchange| as thresholds for significant differential expression. The enrichment analysis was performed based on the hypergeometric test. For KEGG, the hypergeometric distribution test was performed as a pathway. For GO, it was performed based on the GO term.

### 2.5 Targeted metabolomics analysis of phytohormones

#### 2.5.1 Extraction of phytohormones from ginseng hairy roots

The hairy root was ground at a low temperature (30 Hz, 1 min). We weighed 50 mg of the ground hairy root and added 10 μL of mixed internal standard solution (100 ng/mL). Then, 1 mL of methanol/water/formic acid (15:4:1, v/v/v) was added, vortexed, and shaken for 10 min. The mixture was centrifuged at 12,000 rpm under 4 °C for 5 min. We took the supernatant, concentrated it to dryness, and redissolved it with 100 μL 80% methanol. It was filtered with 0.22 μm Millipore for UPLC-MS/MS analysis.

#### 2.5.2 Detection of phytohormones by UPLC-MS/MS

Ultra-high performance liquid chromatography-triple quadrupole ion trap mass spectrometer (QTRAP 6500+, AB SCIEX) and Waters ACQUITY UPLC HSS T3 C18 column (100 mm × 2.1 mm, 1.8 µm) were used to detect phytohormones.

Chromatographic conditions: mobile phase: 0.04% acetic acid in water (A) and 0.04% acetic acid in acetonitrile (B); gradient elution: 0–1 min 5% B, 1–8 min 5%–95% B, and 8–9 min 95% B; flow rate: 350 μL/min; column temperature: 40 °C; injection volume: 2 μL.

Mass spectrometry conditions: electrospray ionization (ESI) in both positive and negative ion modes. Ion spray voltage: 5500 V (positive) and −4500 V (negative); source temperature: 550°C; curtain gas: 35 psi. Phytohormones were analyzed using scheduled multiple reaction monitoring (MRM). Mass spectrometer parameters including the declustering potentials (DP) and collision energies (CE) for individual MRM transitions were done with further DP and CE optimization.

#### 2.5.3 Data analysis

Data acquisitions were performed using Analyst 1.6.3 software (Sciex). Multiquant 3.0.3 software (Sciex) was used to quantify all metabolites. The profiles of phytohormones were compared and the contents were analyzed by orthogonal partial least square discriminant analysis (OPLS-DA) using SIMCA-P Statistics 13.0 software. A *t*-test was used to assess the significant difference (*p <* 0.05) using SPSS. The enrichment analysis of metabolic pathways was carried out using the KEGG (http://www.kegg.com/) database.

## 3 Results

### 3.1 The changes of ginsenosides during ginseng decomposition in soil

**FIGURE 1:**
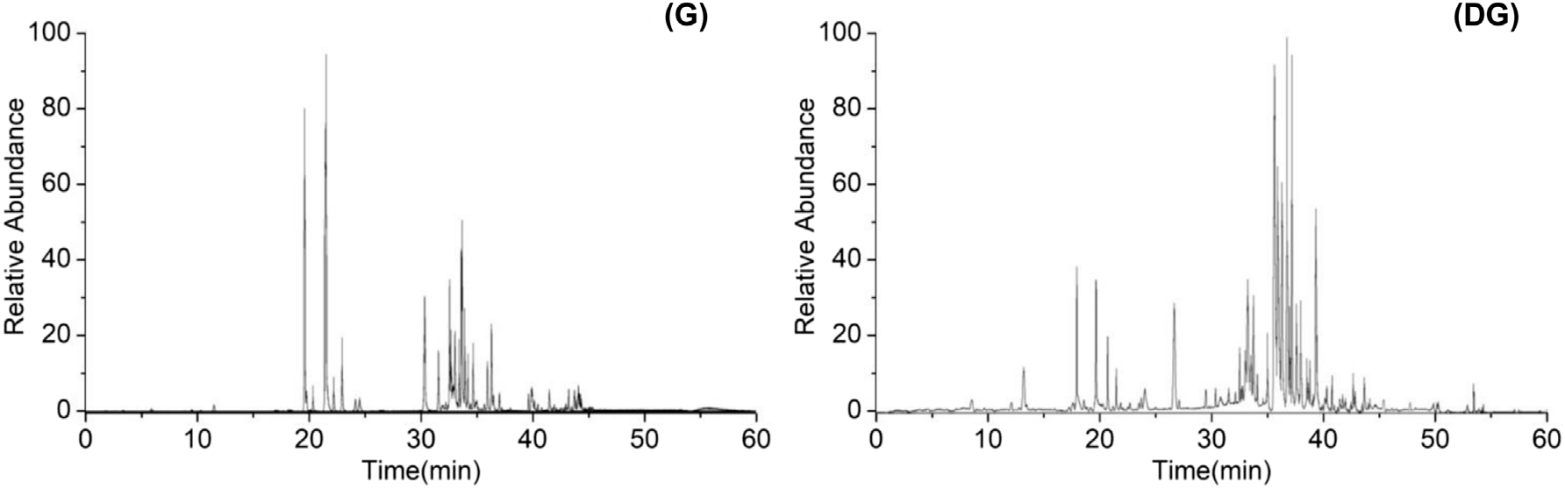
Total ion current chromatogram of ginsenosides in ginseng (G) and decomposed ginseng (DG).

Ginsenosides in ginseng and its soil decompositions were determined by UPLC-HRMS. Figure 1 shows the total ion current chromatogram (TIC) of ginsenosides in negative ion mode. The relative retention time, precise molecular weight in MS, and the fragment ions in MS^2^ of the compounds detected were compared with the ginsenosides standard and data reported in the literature (Zhang et al., 2016; Chen et al., 2020). A total of 51 ginsenoside components were identified and deduced, as shown in Table 1.

**TABLE 1:** Relative peak area percentages of ginsenosides before and after ginseng decomposition.

tR	[M-H]^-^	Molecular	Compounds	Type	Pre-	Post
		mass			Decomposition	Decomposition
19.85	931.5272	932.5345	NG-R_1_-iso	PPT	0.23	0.06
19.86	961.5378	962.5450	20-Glc-Rf	PPT	1.98	0.68
20.44	931.5272	932.5345	NG-R_1_	PPT	1.30	0.53
21.54	945.5428	946.5501	Re	PPT	15.98	13.20
21.58	799.4849	800.4922	Rg_1_	PPT	21.35	10.44
22.78	801.5006	802.5079	Rf_2_	PPT	4.90	0.00
23.04	885.4853	886.4926	Malonyl-Rg_1_	PPT	0.53	0.00
23.04	841.5400	842.5400	Acetyl-Rg_1_-iso	PPT	0.02	0.00
24.59	885.5500	886.4926	Malonyl-Rg_1_-iso	PPT	0.23	0.00
24.59	841.5400	842.5400	Acetyl-Rg_1_	PPT	1.18	0.00
29.42	783.4900	784.4973	20(S)-Rg_2_	PPT	3.07	1.85
30.40	799.4849	800.4922	20(S)-Rf	PPT	5.65	4.87
31.45	799.4849	800.4922	20(R)-Rf	PPT	1.01	0.01
32.32	1209.6200	1210.6346	Ra_1_	PPD	0.52	0.27
32.37	769.4744	770.4816	F_3_	PPT	N/A	0.03
32.51	1193.5961	1194.6033	Malonyl-Rb_1_	PPD	0.22	0.02
32.53	1239.6379	1240.6452	R_4_	PPD	0.53	0.62
32.54	1325.6383	1326.6456	Malonyl-Ra_3_	PPD	0.02	0.00
32.66	1107.5957	1108.6029	Rb_1_	PPD	4.47	3.47
32.67	621.4372	622.4445	Compound K	PPD	N/A	1.50
32.77	783.4900	784.4973	20(R)-Rg_2_	PPT	0.02	0.00
32.79	825.5006	826.5079	20(S)-acetyl-Rg_2_	PPT	0.22	0.02
32.93	769.4744	770.4816	NG-R_2_	PPT	0.08	0.05
32.93	825.5006	826.5079	20(R)-acetyl-Rg_2_	PPT	0.50	0.00
33.14	1149.7200	1150.7100	Quinquefolium-R_1_	PPD	0.36	0.07
33.22	637.4321	638.4394	20(S)-Rh_1_	PPT	0.06	1.40
33.40	1209.6200	1210.6346	Ra_2_	PPD	0.62	0.11
33.52	1077.5851	1078.5924	Rc	PPD	5.22	4.56
33.75	955.4908	956.4981	Ro	OLE	0.19	0.10
33.83	1295.6278	1296.6350	Malonyl-Ra_1_	PPD	0.81	0.00
34.01	1119.5957	1120.6029	Rs_1_	PPD	0.00	0.11
34.02	1163.5855	1164.5928	Malonyl-Rc	PPD	6.71	0.00
34.27	1209.6200	1210.6346	Ra_1_-iso	PPD	0.15	0.07
34.30	1077.5851	1078.5924	Rb_2_	PPD	7.59	5.97
34.54	1077.5851	1078.5924	Rb_3_	PPD	1.59	0.49
34.75	1163.5855	1164.5928	Malonyl-Rb_2_	PPD	5.10	0.00
34.76	1119.5957	1120.6029	Rs_2_	PPD	0.00	0.52
35.08	1163.5855	1164.5928	Malonyl-Rb_3_	PPD	1.01	0.00
35.20	621.4372	622.4445	20(S)-Rh_2_	PPD	N/A	0.11
35.75	637.3539	638.4394	20(R)-Rh_1_	PPT	N/A	1.08
36.03	945.5428	946.5501	Rd	PPD	4.54	20.52
36.41	987.5200	988.5200	Pseudo-Rc_1_	PPD	0.37	0.07
37.00	945.5428	946.5501	Gypenoside X Ⅶ	PPD	0.07	0.73
37.10	1031.5432	1032.5505	Malonyl-Rd	PPD	1.00	0.00
37.68	621.4372	622.4445	20(R)-Rh_2_	PPD	N/A	5.00
38.85	637.4321	638.4394	F_1_	PPT	0.00	1.06
39.44	783.4900	784.4973	F_2_	PPD	0.00	1.52
39.44	825.5006	826.5079	20(S)-Rs_3_	PPD	N/A	0.05
39.69	825.5006	826.5079	20(R)-Rs_3_	PPD	N/A	0.97
39.72	783.4900	784.4973	20(S)-Rg_3_	PPD	0.00	3.69
40.43	783.4900	784.4973	20(R)-Rg_3_	PPD	N/A	3.01

The relative percentage contents of 51 ginsenosides were calculated and compared. The results showed that the contents of PPD-type ginsenosides Ra_1_, Rb_1_, Quinquefolium-R_1_, Ra_2_, Rc, Ra_1_-iso, Rb_2_, Rb_3_, and Pseudo-Rc_1_ were reduced after decomposition. Ginsenosides R_4_, Rs_1_, Rs_2_, Rd, Gypenoside X Ⅶ, F_2_, and 20(*S*)-Rg_3_ increased in content. Ginsenosides 20(*S*)-Rh_2_, 20(*R*)-Rh_2_, 20(*S*)-Rs_3_, 20(*R*)-Rs_3_, 20(*R*)-Rg_3_, and Compound K were produced. PPT-type ginsenosides NG-R_1_-iso, 20-Glc-Rf, NG-R_1_, Re, Rg_1_, Rf_2_, 20(*S*)-Rg_2_, 20(*R*)-Rg_2_, 20(*S*)-Rf, 20(*R*)-Rf, and NG-R_2_ were reduced after decomposition, while ginsenosides 20(*S*)-Rh_1_ and F_1_ increased. Ginsenosides F_3_ and 20(*R*)-Rh_1_ were produced. OLE-type ginsenoside Ro was decreased by decomposition. Malonyl and acetyl-substituted ginsenosides were all reduced as desubstituent transformation.

It has been reported that ginsenosides with high molecular weight and high polarity were converted to rare ginsenosides with low molecular weight and low polarity by hydrolysis of glycosidic bonds (Kim et al., 2012; Palaniyandi et al., 2016; Huang et al., 2019; Li et al., 2023). The results of this study showed that the transformation of ginsenosides by microorganisms in soil was in agreement with the reported findings. It was possible to deduce the decomposed biotransformation pathways of PPD and PPT-type ginsenosides. As shown in Supplementary Figure S1, the conversion pathways of PPD-type ginsenoside were Rb_1_, Rb_2_, Rb_3_, Rc→Rd→F_2_+Rg_3_→Rh_2_. The conversion pathways of PPT-type ginsenoside were Re→Rg_2_→Rh_1_, Re→F_1_, Rg_1_→F_1_+Rh_1_.

### 3.2 Validation of allelopathic autotoxicity of decomposed ginseng

#### 3.2.1 Apparent morphological changes and proliferative multiples of ginseng hairy roots

The growth of ginseng hairy roots was relatively slow on days 1–14, and more rapid on days 15–20, which was the optimal growth period. After 20 days, the growth was in a period of relative stability with basically no increase in weight. Compared with group C, groups G and DG gradually showed serious growth stagnation after the rapid growth period. The monthly proliferative multiple was calculated with the formula: monthly proliferative multiple=(harvest amount−inoculum amount)/inoculum amount × 100%. As shown in Supplementary Figure S2, the proliferative multiples of ginseng hairy root growth gradually decreased with the increase of ginsenosides’ concentration. The smallest proliferative multiple of ginseng hairy root growth was observed when 10 mg/L ginsenosides groups were added. The growth inhibition effect was more significant in the DG group than in the G group.

**FIGURE 2:**
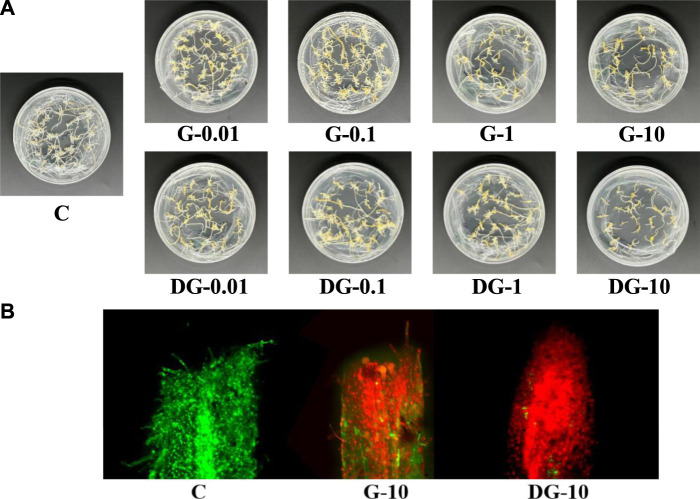
Apparent morphology of ginseng hairy root in C, G, and DG groups **(A)**. FDA-PI fluorescence staining of ginseng hairy roots in C, G, and DG groups **(B)**.

For the apparent morphological determination, the influence of ginsenoside extracts on the growth of ginseng hairy roots was compared and shown in Figure 2A. The overall growth state of hairy roots in group C was good, longer, and thicker, as well as having a yellow color and many branches. Compared to group C, the growth of ginseng hairy root was significantly inhibited in both groups G and DG. With the increase of ginsenoside concentration, the inhibitory effect increased gradually. The hairy root gradually changed from a light yellow color with many branches and dense growth to a dark yellow color with few branches and sparse growth. Therefore, the growth state showed an apoptotic tendency. With an addition of 10 mg/L ginsenosides, the growth of hairy roots was almost shriveled in both the G and DG groups. Compared to the G group, the overall growth of hairy roots in the DG group was sparser and more shriveled with fewer branches. It means that the growth of hairy roots in the DG group was inhibited more severely. The results showed that the ginsenosides extracts had significant inhibitory effects on the growth of ginseng hairy roots, and the inhibitory effect of the rare ginsenosides in decomposed ginseng was stronger.

#### 3.2.2 The influence on apical cell viability of ginseng hairy roots

According to the observation of the apparent morphology of ginseng hairy roots, the G-10 and DG-10 groups presented the strongest inhibitory effects on their growth. Furthermore, the apical cell viability in these two groups was determined using confocal laser scanning microscopy. The localization of live and dead cells in the apical root was observed and is shown in Figure 2B. The apical cells were in good condition in group C. The G-10 and DG-10 groups showed different degrees of cell apoptosis. The apical cells were damaged, and the damage was more serious in the DG-10 group. It indicated that both prototype and rare ginsenosides had inhibitory effects on the apical cell of the hairy root, and the inhibitory effect of rare ginsenosides was stronger. This result was consistent with the apparent morphological changes of ginseng hairy roots.

### 3.3 Transcriptomics of allelopathic mechanisms of ginseng decomposition

#### 3.3.1 Gene functional annotation

**FIGURE 3:**
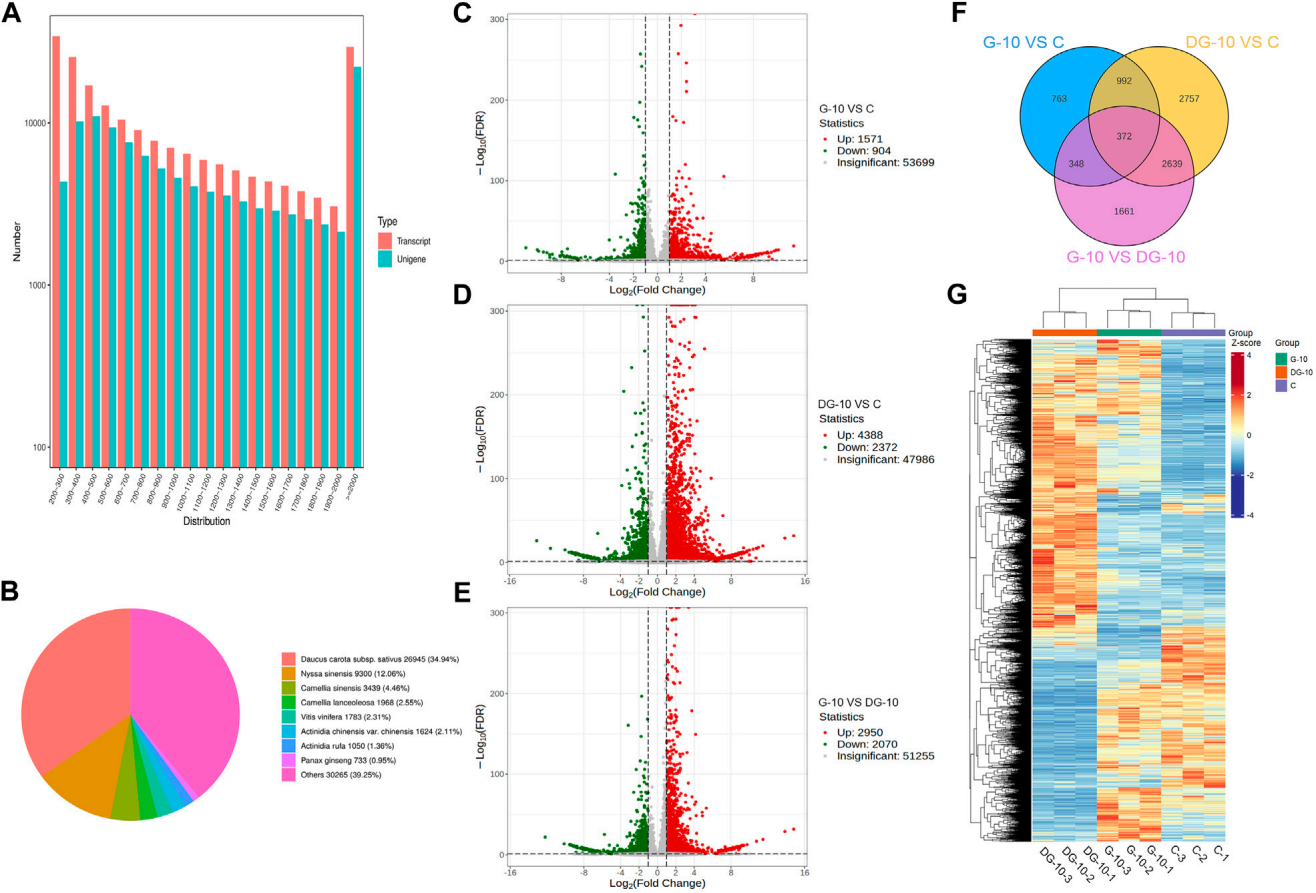
Sequence length distribution map of unigenes **(A)**. Non-redundant homologous species distribution **(B)**. Volcano map of DEGs screening for groups G-10 vs. C **(C)**, DG-10 vs. C **(D)**, and G-10 vs. DG-10 **(E)**. Venn diagram of DEGs in ginseng hairy root between groups **(F)**. Clustering heat map of DEGs in ginseng hairy roots between groups **(G)**.

A reference-free transcriptome was used for comparative analysis. For subsequent analysis, the transcript sequences obtained from Trinity splicing were used as reference sequences. The longest cluster sequence obtained from Corset hierarchical clustering was used as the unigenes. The length distribution of the sequence is shown in Figure 3A. The unigenes with sequence lengths greater than or equal to 2000 nt were the most abundant.

The unigenes sequences were aligned with KEGG, NR, Swiss-Prot, TrEMBL, COG/KOG, and GO databases using DIAMOND BLASTX software and aligned with the Pfam database using HMMER software. The annotation information of unigenes was obtained. A total of 111,001 unigenes were annotated into the database, with an annotation rate of 100%. Among them, 59,581 unigenes were annotated into the KEGG database (53.68%), 77,107 unigenes were annotated into the NR database (69.47%), 58,346 unigenes were annotated into the Swiss Prot database (52.56%), 76,893 unigenes were annotated into the TrEMBL database (69.27%), 47,116 unigenes were annotated into the KOG database (42.45%), 68,042 unigenes were annotated into the GO database (61.30%), and 45,836 unigenes were annotated into the Pfam database (41.29%). Comparative analysis showed that the most unigenes were annotated into the NR database and the least unigenes were annotated into the Pfam database.

The annotation results of the NR database are shown in Figure 3B. The most annotated species was carrot (Daucus carota) with a total of 26,945 unigenes (approximately 34.94%), and the other homologous species were Nyssa sinensis (12.06%), Camellia sinensis (4.46%), Camellia lanceoleosa (2.55%), Vitis vinifera (2.31%), Actinidia chinensis var. chinensis (2.11%), and Actinidia rufa (1.36%). This result showed that the hairy root tissue of ginseng has the greatest homology with carrot.

#### 3.3.2 Screening for differentially expressed genes (DEGs)

To explore the allelopathic mechanism of ginsenosides on hairy roots before and after ginseng decomposition, genes in the transcriptome library were analyzed for differential comparison, and DEGs were obtained. Using DESeq2, an analysis of DEGs between two groups was performed. As shown in Figures 3A, C total of 2,745 DEGs were obtained in the comparison of groups G-10 vs. C, of which 1,571 genes were upregulated and 904 genes were downregulated, significantly. As shown in Figures 3A, D total of 6,760 DEGs were obtained between groups DG-10 vs. C, of which 4,388 genes were significantly upregulated and 2,372 genes were significantly downregulated. In Figures 3A, E total of 5,020 DEGs were obtained between groups G-10 vs. DG-10, of which 2,950 genes were significantly upregulated and 2070 genes were significantly downregulated. The Venn diagram of the screened DEGs is shown in Figure 3F, which shows the overlap between groups. The hierarchical cluster analysis of DEGs in each group is shown in Figure 3G. The screened DEGs could reflect the intergroup differences between the samples. Both prototype and rare ginsenosides affected the expression of genes in ginseng hairy roots, and there was a significant difference in the expression of related genes between prototype and rare ginsenosides. Ginsenosides may be one of the main factors affecting the growth and development of ginseng hairy roots.

#### 3.3.3 Enrichment analysis of pathways of DEGs

**FIGURE 4:**
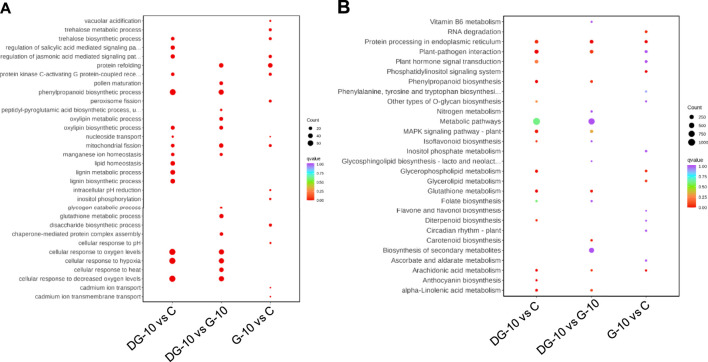
Enrichment plots of the number of DEGs analyzed by GO **(A)** and KEGG **(B)** analysis.

The enrichment of pathways of DEGs in ginsenoside-treated hairy roots was analyzed using GO and KEGG databases. The results of GO enrichment of the DEGs are shown in Figure 4A. In groups G-10 vs. C, the DEGs were mainly involved in protein refolding, protein kinase C-activating G protein-coupled receptor signaling, trehalose biosynthetic process, and mitochondrial and peroxisome fission pathways. The DEGs of the DG-10 vs. C groups were mainly involved in cellular response to hypoxia, oxygen levels, decreased oxygen levels, and phenylpropanoid biosynthetic process. The DEGs of G-10 vs. DG-10 groups were mainly involved in cellular response to oxygen levels, decreased oxygen levels, hypoxia, and heat-shock protein binding.

The results of KEGG enrichment of DEGs are shown in Figure 4B. The DEGs in groups G-10 vs. C and DG-10 vs. C were mainly involved in the biosynthesis of secondary metabolites and metabolic pathways and phytohormone signal transduction. The DEGs in groups G-10 vs. DG-10 were mainly involved in the biosynthesis of secondary metabolites and metabolic pathways and protein processing in the endoplasmic reticulum. The DEGs in the three groups’ comparison were mainly involved in the biosynthesis of secondary metabolites and metabolic pathways.

### 3.4 Targeted metabolomics of endogenous phytohormones in ginseng hairy roots

#### 3.4.1 UPLC-MS/MS analysis of endogenous phytohormones

According to the results of allelopathic autotoxicity validation, the G-10 and DG-10 groups presented the most significant activities. Therefore, the 109 endogenous phytohormones in the ginseng hairy roots of these two groups were analyzed by UPLC-MS/MS. A total of 53 phytohormones were detected, and the contents are shown in Supplementary Table S1. The changes of these endogenous phytohormones between G-10 and DG-10 groups were further analyzed by targeted metabolomics strategy.

#### 3.4.2 Screening for differential metabolized phytohormones (DMPs)

**FIGURE 5:**
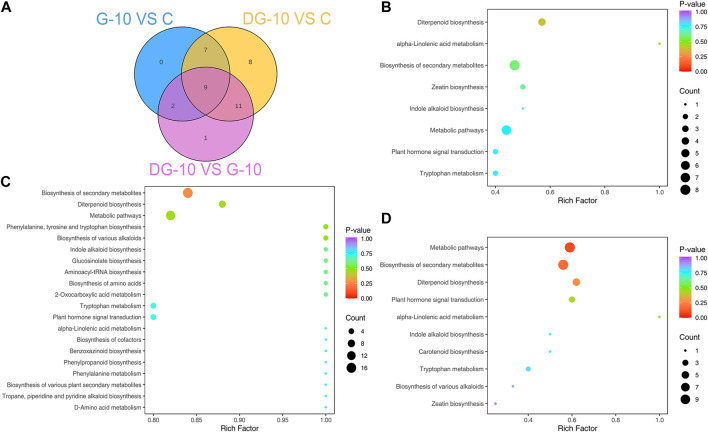
Venn diagram of DMPs in ginseng hairy root between groups **(A)**. Enrichment plots of metabolic pathways of DMPs for groups G-10 vs. C **(B)**, groups DG-10 vs. C **(C)**, and groups G-10 vs. DG-10 **(D)**.

Univariate statistical analysis and multivariate statistical analysis were applied to screen DMPs in ginseng hairy roots, as shown in Table 2. The comparison of DMPs between groups was visible in the Venn diagram (Figure 5A). A total of 18 DMPs were screened out in groups G-10 vs. C. A total of 35 DMPs were screened out between groups DG-10 and C. For groups G-10 vs. DG-10, 23 DMPs were screened out. Among the three groups, a total of 9 DMPs (IAM, TRA, ICA, 2MeScZR, cZRMP, GA53, GA19, JA, and JA-Val) were screened out.

**TABLE 2:** The DMPs in ginseng hairy roots screened by metabolomics.

Compounds	Class	G-10 vs. C	DG-10 vs. C	G-10 vs. DG-10
ABA	ABA	-	up	up
TRP	Auxin	-	up	-
IAM	Auxin	up	up	up
TRA	Auxin	up	up	up
OxIAA	Auxin	-	up	up
IAA-Trp	Auxin	-	up	-
IAA-Glu	Auxin	-	up	-
IAA-Glc	Auxin	-	down	down
ILA	Auxin	-	up	-
ICA	Auxin	up	up	up
IAA-Phe	Auxin	up	up	-
IAA-Asp	Auxin	-	up	up
2MeScZR	CK	up	up	up
cZRMP	CK	up	up	up
tZRMP	CK	-	-	up
cZ	CK	-	up	up
cZR	CK	up	up	-
IP	CK	-	up	-
tZR	CK	down	down	-
GA53	GA	up	up	up
GA19	GA	up	up	up
GA12-ald	GA	-	up	-
GA8	GA	-	down	-
GA9	GA	-	up	up
GA20	GA	down	down	-
GA24	GA	up	-	down
GA7	GA	-	up	up
OPC-4	JA	-	up	up
JA	JA	up	up	up
JA-ILE	JA	up	up	-
JA-Val	JA	up	up	up
H2JA	JA	up	up	-
12-OH-JA	JA	up	-	down
OPC-6	JA	up	up	-
Phe	SA	-	up	-
SA	SA	-	up	up
SAG	SA	-	up	up

#### 3.4.3 Enrichment analysis of metabolic pathways of DMPs

The enrichment analysis of metabolic pathways of DMPs was performed based on the KEGG database. For groups G-10 vs. C, the metabolism of DMPs was mainly involved in eight pathways (Figure 5B), biosynthesis of secondary metabolites, diterpenoid, zeatin and metabolic pathways, phytohormone signal transduction, and tryptophan metabolism. In groups DG-10 vs. C, the DMPs were with significant enrichment in the biosynthesis of secondary metabolites, diterpenoid and metabolic pathways, tryptophan metabolism, and phytohormone signal transduction (Figure 5C). DMPs were mainly involved in 10 pathways in groups G-10 vs. DG-10, with significant enrichment also in the biosynthesis of secondary metabolites, diterpenoid and metabolic pathways, phytohormone signal transduction, and tryptophan metabolism (Figure 5D).

### 3.5 Integrated targeted transcriptomics and metabolomics analysis of allelopathic mechanisms of ginseng decomposition

#### 3.5.1 Enrichment analysis of pathways of DMPs and DEGs

**FIGURE 6:**
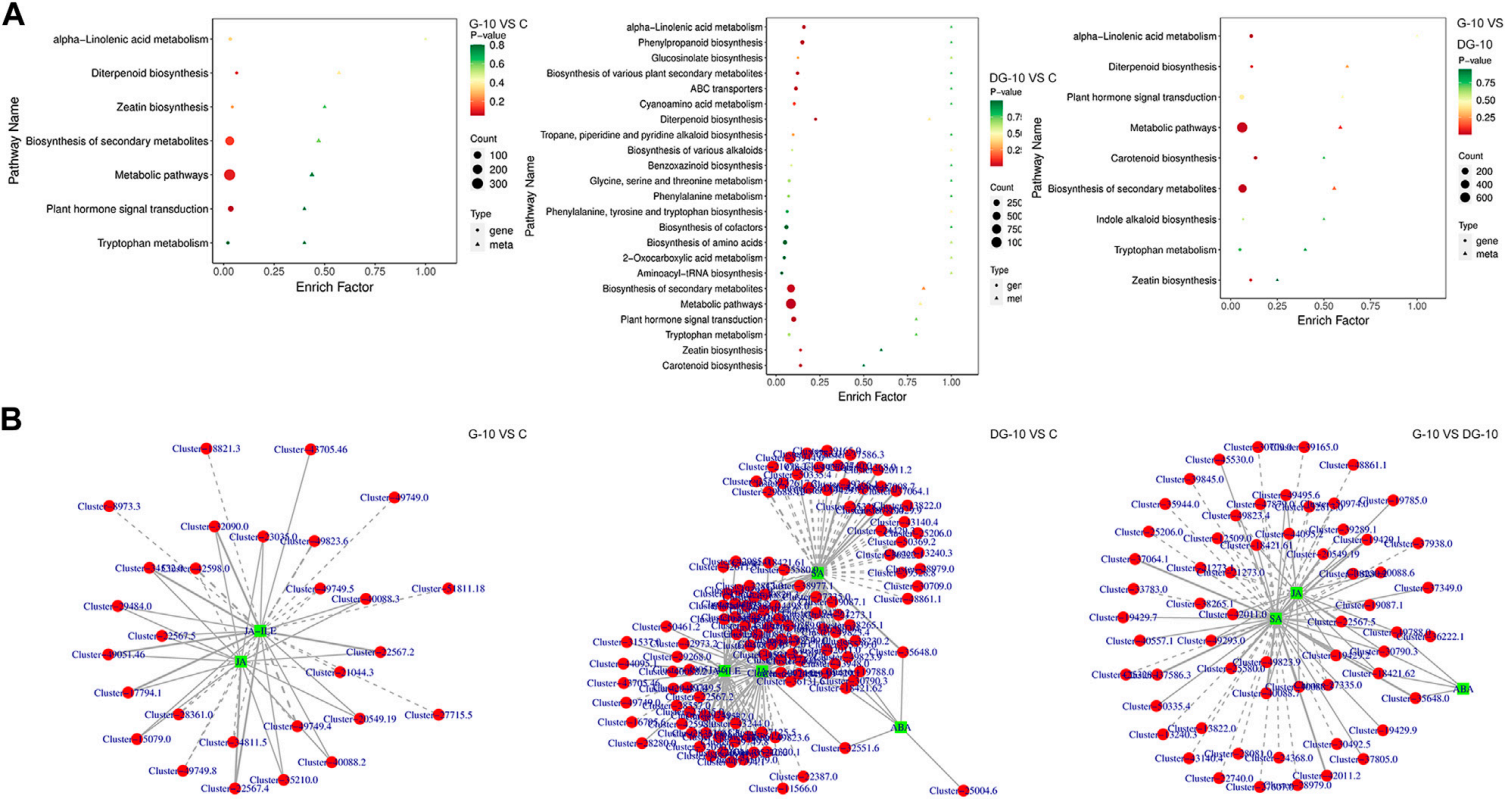
Enrichment plots of DEGs and DMPs by KEGG analysis **(A)**. Network diagram of DEGs and DMPs correlation **(B)**.

An integrated analysis of targeted transcriptomics of DEGs and metabolomics of DMPs was applied. The KEGG enrichment of pathways of the DEGs and DMPs is shown in Figure 6A. In groups G-10 vs. C, 7 pathways were enriched. In groups DG-10 vs. C, 23 pathways were enriched. In groups G-10 vs. DG-10, 9 pathways were enriched. For the three groups’ analysis, biosynthesis of secondary metabolites, diterpenoid and zeatin, metabolic pathways, alpha-linolenic acid, tryptophan metabolism, and phytohormone signal transduction were enriched. Furthermore, phytohormone signal transduction pathways were selected for correlation analysis.

#### 3.5.2 Correlation analysis of gene expression and phytohormone metabolism

By correlation analysis, the DMPs and DEGs in the phytohormones signal transduction pathways with Pearson correlation coefficients greater than 0.80 and *p*-values less than 0.05 were selected. The network diagram of the correlation of DMPs and DEGs was constructed and shown in Figure 6B. A total of 53 DEGs and two DMPs were enriched in groups G-10 vs. C, 152 DEGs and four DMPs were enriched in groups DG-10 vs. C, and 90 DEGs and three DMPs were enriched in groups G-10 vs. group DG-10. It was observed that genes and phytohormones in ginseng hairy roots showed different accumulation patterns after the treatment of prototype and rare ginsenosides. These DEGs may be the key genes regulating the accumulation of DMPs in ginseng hairy roots. The prototype and rare ginsenosides intervened in the growth and development of ginseng hairy roots through the phytohormones signal transduction pathways.

#### 3.5.3 Pathways analysis of gene expression and phytohormone metabolism

**FIGURE 7:**
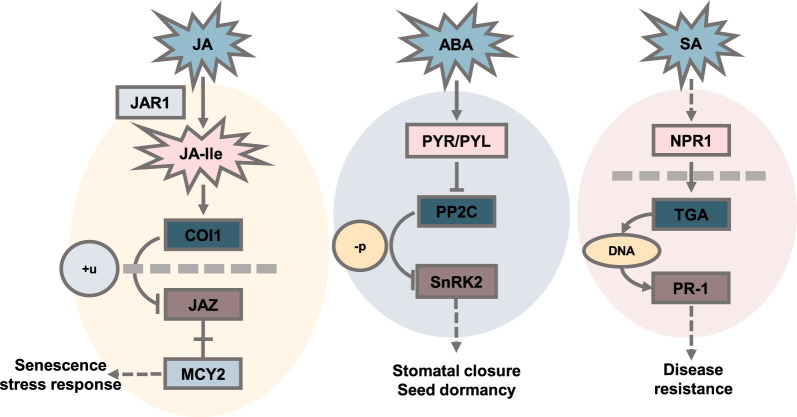
The synthesis signaling pathways of JA, ABA, and SA.

Based on the results of integrated analysis, the relevant phytohormones and genes in the phytohormones signal transduction pathways were simultaneously mapped onto the KEGG pathway, as shown in Figure 7.

The prototype ginsenosides in ginseng were able to upregulate the gene expression of JAR1, COI1, and JAZ in the JA synthesis pathway. Whereas, MCY2 had both upregulated and downregulated gene expression. JA through JAR1 was able to promote the synthesis of JA-Ile, which acted as an activator that would promote the interaction between JAZ and COI1. COI1 inhibited the gene expression of JAZ through the ubiquitination pathway, thereby promoting JAZ degradation. As a result of JAZ degradation, it was able to control the upregulation of JAZ transcription factors, thus affecting the growth and development of plants and the tolerance to biotic and abiotic stresses. Subsequently, JAZ dissociated MCY2, which in turn interacted with DNA to participate in senescence and stress response processes. The synthesis of JA and JA-Ile could be promoted by gene expression of JAR1, COI1, JAZ, and MCY2. JA-Ile is one of the derivatives of JA. In plants, they were the key signaling compounds related to stress defense and development and were able to inhibit growth (Mouna Ghorbel). Our result showed that the intervention of prototype ginsenosides promoted the secretion of JA and JA-Ile in ginseng hairy roots, which in turn inhibited the growth and development of hairy roots.

The rare ginsenosides in decomposed ginseng were able to upregulate the expression of some genes of PYR/PYL and PP2C and downregulate the expression of some genes of PYR/PYL, PP2C, and SnRK2 in the ABA synthesis pathway. The accumulation of ABA activated gene expression of PYR/PYL, which inhibited PP2C. PYR/PYL is an ABA receptor that acts at the apex of the negative regulatory pathway and controls ABA signaling by inhibiting PP2C, and PP2C inhibited the expression of SnKR2s through the dephosphorylation pathway and inactivated SnRK2. This signal synthesis pathway was involved in the process of stomatal closure and seed dormancy. The increased accumulation of ABA could impair the growth of plants (Polavarapu B Kavi Kishor).

After ginseng decomposition, the expression of some genes of PR-1 in the SA synthesis pathway was upregulated and the expression of some genes of NPR1 and TGA was downregulated. SA could indirectly promote gene expression of NPR1, which acted as a key regulator of plant species defense-activated gene expression of TGA. TGA indirectly regulated gene expression of PR-1 by interacting with NPR1 and binding to DNA. Activation of these genes promoted the secretion of SA, which resulted in the inhibition of plant growth and development (Bruno Pok Man Ngou).

## 4 Discussion

Ginsenosides are a major kind of allelochemicals in ginseng. With the accumulation of ginsenosides in the soil, the microbial community and metabolic activity in the soil could change. As the microbiology of the soil altered, the growth of ginseng was affected and continuous cropping obstacles were presented (Zhang et al., 2017). Cheng ZH et al. found that ginsenosides R_1_, Rg_3_, Re, Rb_2_, Rb_1_, Rg_1_, Rg_2_, and Rd could accumulate in the rhizosphere soil through root secretion or decomposition and impede seedling emergence and growth (Cheng et al., 2015). Shen YL et al. found that the ginsenoside Rd possessed significant allelopathic activity on ginseng seed germination, seedling growth, and plant development (Shen et al., 2023). Li YL et al. found that the monomer ginsenosides Rg_1_, Rb_1_, Rh_1_, and their mixtures were able to disrupt the balance of fungal microbiome by stimulating potential soil-borne pathogens, leading to replanting failure of Panax notoginseng. They demonstrated that the allelopathic autotoxicity of ginsenosides was one of the reasons for continuous cropping obstacles in ginseng planting (Li et al., 2020).

In this study, prototype ginsenosides were converted into rare ginsenosides after ginseng decomposition by soil microorganisms. Both prototype and rare ginsenosides had a significant inhibitory effect on the growth of ginseng. The newly generated rare ginsenosides through decomposition had a more significant inhibitory effect on the growth of ginseng, which showed that rare ginsenosides had stronger allelopathic autotoxicity. It was reported that the relative content of prototype ginsenosides (Rb_1_, Rb_2_, and Rc) decreased, which was secreted in the rhizosphere soil by the ginseng root system, while the relative content of degraded ginsenoside products (Rd, G-VXII, and F_2_) increased in rhizosphere soil during ginseng growth (Li et al., 2021). Some studies showed that secretions of ginseng root could mediate negative plant-plant interactions and lead to stronger allelopathy (Cheng et al., 2015; Ding et al., 2019; Feng et al., 2022). It was demonstrated that rare ginsenosides were released through root secretion and decomposition and were then accumulated in the soil.

Phytohormones such as growth hormone, cytokinin, gibberellin, abscisic acid, ethylene, jasmonic acid, and salicylic acid played important roles in plant growth and stress responses (Zhao et al., 2023). Allelochemicals might interfere with plant growth by regulating the metabolic levels of phytohormones. Disturbances in phytohormone levels resulted in reduced metabolic activity of embryos and hindered their germination and growth (Bogatek and Gniazdowska, 2014). In this study, we found that ginsenosides could significantly affect the accumulation metabolism of endogenous phytohormones in ginseng hairy roots. The endogenous phytohormones played an important regulatory role in the allelopathic autotoxicity of ginsenosides. Some studies reported that the imbalance of accumulation of Auxin was one of the causes of allelopathic autotoxicity (Zhang et al., 2018). Accumulation of JA triggered the overactivation of defense mechanisms, which affected plant growth and development (Li et al., 2022). Peng YJ et al. found that low concentration of SA treatment stimulated the growth of some plant species, and excessive accumulation of SA inhibited plant growth and development (Peng et al., 2021). Brookbank B. P. found that ABA promoted adaptive responses to abiotic and biotic stresses, and under stress, ABA levels increased sharply and resulted in inhibition of germination and growth stagnation (Brookbank et al., 2021).

Integrated analysis of transcriptomics and metabolomics can provide more insight into biological mechanisms than these two methods applied individually (Li et al., 2016). An increasing number of studies applied combined transcriptomics and metabolomics analysis methods to correlate gene expression and metabolites to explain biological mechanisms much better (Zhang et al., 2021; Wan et al., 2022; Wu et al., 2023). In this study, we analyzed the phytohormones JA, ABA, and SA signaling pathways using integrated analysis of transcriptomics and targeted metabolomics. The allelopathy of prototype and rare ginsenosides as well as the allelopathic mechanisms of ginseng decomposition were investigated.

JA and its derivatives were able to regulate a wide range of biological processes in plant defense, growth control, and reproductive development. JA-Ile was a major form of JA-amino acid coupler that could be synthesized by recombinant JAR1 (Guranowski et al., 2007; Kitaoka et al., 2014). JAR1 was an enzyme that coupled JA to isoleucine, which was shown to play a role directly in COI1-mediated signaling (Suza and Staswick, 2008). COI1 was a key participant in the downstream process of JA biosynthesis. JA-Ile bonded to COI1 and promoted the formation of the COI1-JAZ complex, leading to the ubiquitination and subsequent degradation of JAZ (Gfeller et al., 2010). The JAZ family were transcriptional repressors of the jasmonate-responsive. The discovery of JAZ deterrents defined the core JA signaling module as COI1-JAZ-MYC2, and it allowed for a comprehensive understanding of the JA signaling pathway from phytohormone perception to transcriptional reprogramming. Upon phytohormone perception, the JAZ deterrent was degraded by the proteasome, and it released MYC2 and allowed the activation of the JA response (Chini et al., 2009). In this study, the expression of JAR1, COI1, JAZ, and MYC2 genes ultimately led to the accumulation of JA, which inhibited ginseng hairy roots’ growth and development. The JA and SA signaling pathways might activate the release of allelochemicals, as reported by Bi HH et al.

ABA is a key phytohormone for plant stress signaling. Several proteins that participated in the ABA signaling pathway had been identified, such as ABA receptors (PYR/PYL/RCAR), co-receptors PP2Cs (protein phosphatases), SnRK2 kinases (SNF1-related protein kinases), and ABI5/ABFs (transcription factors). In the ABA signal, PYR/PYL receptors interacted with and recruited PP2Cs and then released SnRK2s kinases from sequestration with PP2Cs. This allowed SnRK2s to activate downstream transcription factors of the ABA pathway (Ali et al., 2020). This signaling pathway promoted the expression of key genes in the biosynthesis of PYR/PYL, PP2C, and SnRK2s, which ultimately led to the accumulation of ABA. Furthermore, ABA affected the growth and development of ginseng hairy roots.

NPR1, an SA receptor, plays a key regulatory role in plant immune response (Ji et al., 2022). NPR1 is required for SA-mediated growth inhibition, suggesting a regulatory link between TGA factors and PR gene expression. It plays a role in the PR gene-induced SA signaling pathway. Since NPR1 does not bind to DNA, it may act through one or more transcription factors that mediate the expression of target PR genes. TGA factors play a regulatory role in PR defense gene expression through promoter-specific recruitment mediated by NPR1. TGA factors may also indirectly regulate PR gene expression by interacting with NPR1 to upregulate the expression of other DNA-binding transcription factors (Johnson et al., 2003; Yu et al., 2022). The WRKY transcription factors of the TGA family and alkaline leucine zipper proteins regulate the PR-1 promoter by binding to specific cis-elements (Pape et al., 2010). NPR1, TGA, and PR-1 gene expression of this signaling pathway induced the accumulation of SA, which affected the growth and development of ginseng hairy roots.

## 5 Conclusion

As the decomposition of ginseng root residues in soil, its ginsenoside fractions were microbially transformed to rare ginsenosides and released into the soil. This could aggravate the allelopathic autotoxicity of ginsenosides. Transcriptomics studies showed that ginsenosides were able to significantly affect the expression of genes in the biosynthesis of secondary metabolites and metabolic pathways. Targeted metabolomics studies showed that the intervention of ginsenosides significantly affected the metabolic accumulation of endogenous phytohormones in ginseng hair roots. Integrated analysis of transcriptomics and metabolomics further revealed that ginsenosides significantly affected the DMPs and DEGs in phytohormone signal transduction. Ginsenosides presented allelopathic autotoxicity by intervening in the accumulation of JA, and decomposition enhanced the allelopathic autotoxicity by interfering with the accumulation of ABA and increasing the accumulation of SA in plants. The expression of JAR1, COI1, JAZ, MYC2, PYR/PYL, PP2C, SnRK2s, NPR1, TGA, and PR-1 genes regulated the accumulation of JA, ABA, and SA. It indicated that these DEGs were important potential candidate genes involved in continuous cropping obstacles of ginseng. The results of this study provided a useful reference for in-depth research on resolving continuous cropping obstacles in ginseng planting.

## Data Availability

The original contributions presented in the study are publicly available. This data can be found here: https://www.ncbi.nlm.nih.gov/search/all/?term=PRJNA1068344.
