# Supplementary material for: Exploring the allelopathic autotoxicity mechanism of ginsenosides accumulation under ginseng decomposition based on integrated analysis of transcriptomics and metabolomics
Source: Front Bioeng Biotechnol. 2024 Mar 7;12:1365229. doi: 10.3389/fbioe.2024.1365229 (PMC10955472; doi:10.3389/fbioe.2024.1365229)
Supplement: Supplementary file 1 [file DataSheet1.docx]

Supplementary Material

Exploring the allelopathic autotoxicity mechanism of ginsenosides accumulation under ginseng decomposition based on integrated analysis of transcriptomics and metabolomics

Rui Wang, Tingting Zhou, Yikai Wang, Jinxu Dong, Yutao Bai, Xin Huang*, Changbao Chen*

*** Correspondence:** Xin Huang: huangxinrose@163.com; Changbao Chen: ccb@ccucm.com

**Supplementary Table S1** Endogenous phytohormones in ginseng hairy roots (ng/g**)**

| Index | G-10 Group | DG-10 Group | C Group |
| --- | --- | --- | --- |
| ABA | 2.03 | 9.25 | 1.84 |
| ABA-ald | 29.80 | 58.33 | 33.72 |
| ABA-GE | 11.32 | 17.06 | 11.10 |
| TRP | 8681.45 | 15923.48 | 6712.40 |
| IAM | 0.34 | 0.84 | N/A |
| IPA | N/A | N/A | N/A |
| IBA | N/A | N/A | N/A |
| IAA-Val-Me | N/A | N/A | N/A |
| IAA-Leu-Me | N/A | N/A | N/A |
| IAA-Leu | N/A | N/A | N/A |
| MEIAA | 2.60 | 2.65 | 1.42 |
| IAA-Gly | N/A | N/A | N/A |
| IAA-Glu-diMe | N/A | N/A | N/A |
| IAA-Ala | N/A | N/A | N/A |
| IA | N/A | N/A | N/A |
| TRA | 0.82 | 1.72 | 0.31 |
| OxIAA | 143.40 | 568.93 | 222.21 |
| IAA-Trp | 0.46 | 0.83 | 0.26 |
| IAA-Glu | 1.89 | 3.03 | 1.27 |
| Indole | 1653.22 | 2944.72 | 1177.84 |
| IAA-Glc | 4.12 | N/A | 3.72 |
| ILA | 1.52 | 2.18 | 0.93 |
| ICAld | 5.38 | 9.80 | 5.04 |
| ICA | 1.98 | 4.70 | 0.87 |
| IAA-Phe | 1.35 | 2.06 | 0.07 |
| IAA-Phe-Me | N/A | N/A | N/A |
| IAA-Asp | 192.83 | 507.23 | 96.53 |
| IAA | 25.05 | 33.15 | 24.90 |
| IAA-Val | N/A | N/A | N/A |
| IAN | N/A | N/A | N/A |
| 2MeScZ | N/A | N/A | N/A |
| 2CltZ | N/A | N/A | N/A |
| iP9G | N/A | N/A | N/A |
| oT9G | N/A | N/A | N/A |
| iP7G | N/A | N/A | N/A |
| tZ9G | N/A | N/A | N/A |
| cZROG | N/A | N/A | N/A |
| tZ | N/A | N/A | N/A |
| 2MeScZR | 0.15 | 0.34 | 0.05 |
| 2MeSiPR | N/A | N/A | N/A |
| BAP | N/A | N/A | N/A |
| BAP9G | N/A | N/A | N/A |
| K | N/A | N/A | N/A |
| K9G | N/A | N/A | N/A |
| cZ9G | N/A | N/A | N/A |
| mT | N/A | N/A | N/A |
| mT9G | N/A | N/A | N/A |
| mTR | N/A | N/A | N/A |
| oT | N/A | N/A | N/A |
| oTR | N/A | N/A | N/A |
| pT | N/A | N/A | N/A |
| pT9G | N/A | N/A | N/A |
| pTR | N/A | N/A | N/A |
| 2MeSiP | N/A | N/A | N/A |
| DZ | N/A | N/A | N/A |
| BAP7G | N/A | N/A | N/A |
| tZOG | N/A | N/A | N/A |
| cZRMP | 2.17 | 5.22 | 1.02 |
| tZRMP | 1.35 | 2.99 | 2.16 |
| DHZ7G | N/A | N/A | N/A |
| cZ | N/A | 0.24 | N/A |
| DHZR | 0.18 | 0.17 | 0.18 |
| BAPR | 0.21 | 0.24 | 0.19 |
| cZR | 3.33 | 5.53 | 1.12 |
| iPRMP | 1.00 | 1.21 | 0.78 |
| IPR | 0.31 | 0.28 | 0.19 |
| IP | 0.13 | 0.29 | 0.13 |
| tZR | N/A | N/A | 0.81 |
| DHZROG | 0.09 | 0.15 | 0.11 |
| KR | 0.41 | 0.53 | 0.39 |
| ACC | 74.77 | 55.48 | 87.98 |
| GA3 | N/A | N/A | N/A |
| GA53 | 1.41 | 3.34 | 0.65 |
| GA6 | N/A | N/A | N/A |
| GA19 | 3.99 | 12.25 | 1.73 |
| GA44 | N/A | N/A | N/A |
| GA34 | N/A | N/A | N/A |
| GA12-ald | 0.51 | 0.70 | 0.26 |
| GA5 | N/A | N/A | N/A |
| GA29 | 46.26 | 53.75 | 44.67 |
| GA8 | 4.89 | 2.75 | 5.57 |
| GA1 | N/A | N/A | N/A |
| GA4 | N/A | N/A | N/A |
| GA9 | N/A | 0.83 | N/A |
| GA51 | N/A | N/A | N/A |
| GA20 | N/A | N/A | 0.60 |
| GA24 | 0.27 | N/A | N/A |
| GA7 | N/A | 0.03 | N/A |
| GA15 | 2.28 | 3.18 | 1.82 |
| OPC-4 | N/A | 24.41 | N/A |
| JA | 9.77 | 20.02 | 4.36 |
| JA-ILE | 8.08 | 14.93 | 1.65 |
| JA-Val | 0.13 | 0.30 | 0.04 |
| MEJA | N/A | N/A | N/A |
| JA-Phe | N/A | N/A | N/A |
| JA-ACC | N/A | N/A | N/A |
| OPDA | 2.74 | 3.43 | 1.81 |
| H2JA | 0.18 | 0.23 | N/A |
| 12-OH-JA | 23.52 | N/A | N/A |
| OPC-6 | 63.72 | 108.20 | 14.18 |
| MLT | N/A | N/A | N/A |
| Phe | 2975.33 | 5355.10 | 2446.57 |
| SA | 19.02 | 41.29 | 17.06 |
| SAG | 563.29 | 1881.36 | 503.62 |
| MeSAG | N/A | N/A | N/A |
| 2-Coumarate | N/A | N/A | N/A |
| t-CA | N/A | N/A | N/A |
| ST | N/A | N/A | N/A |
| 5DS | N/A | N/A | N/A |


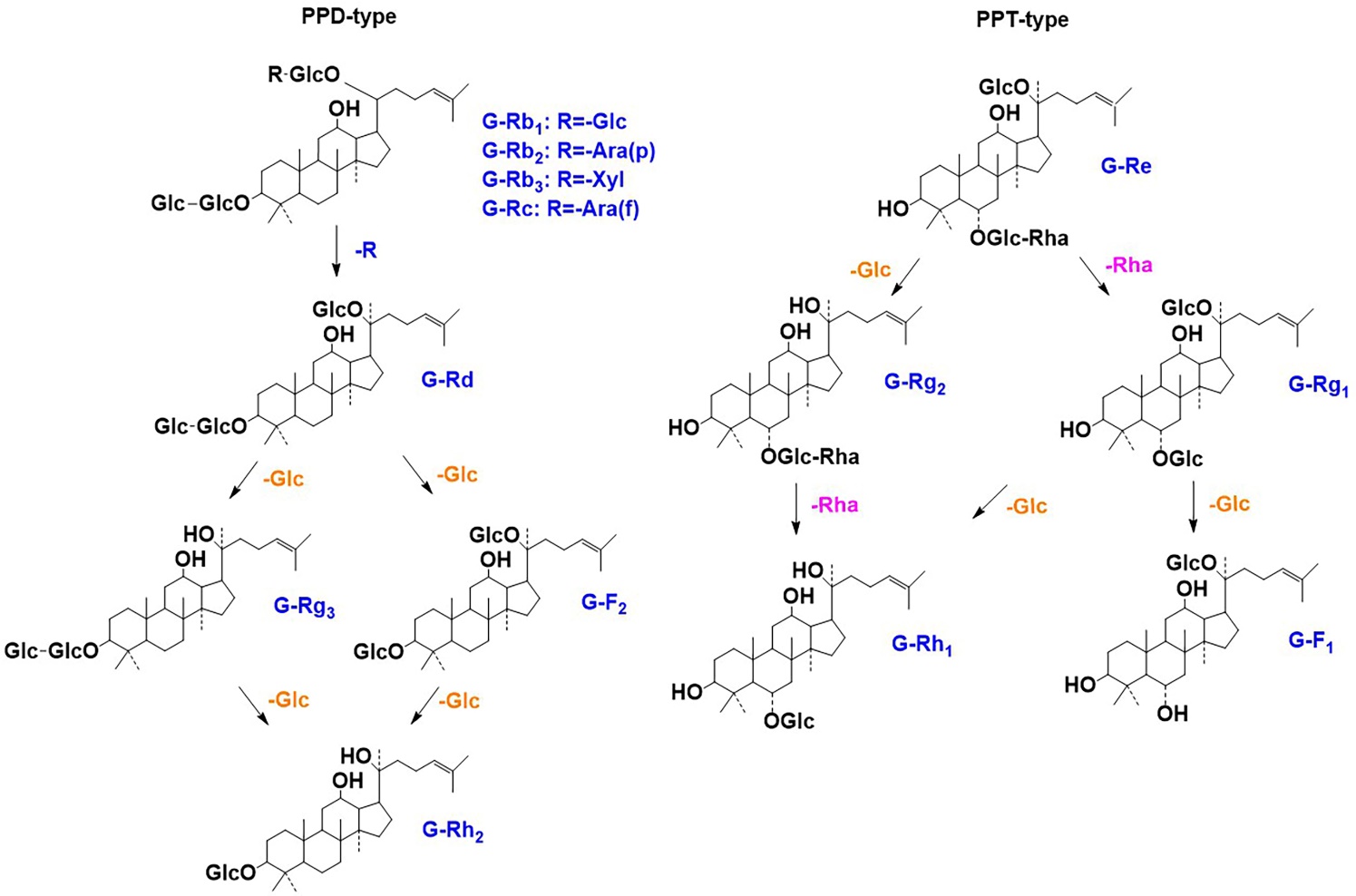


**Supplementary Figure S1** The transformation pathways of PPD-type and PPT-type ginsenosides.


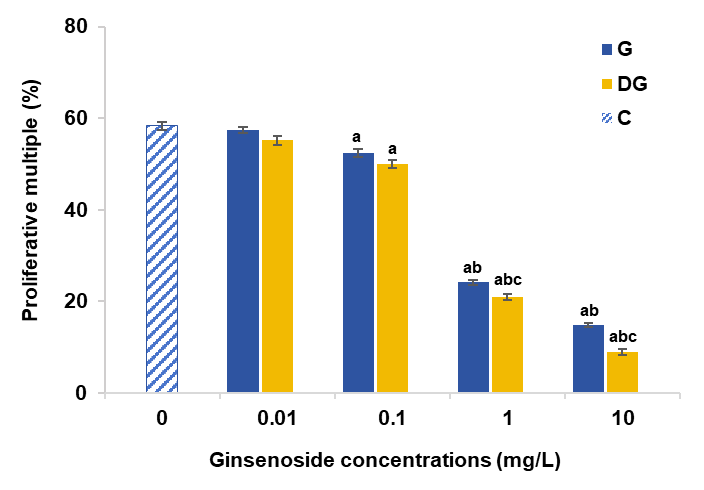


**Supplementary Figure S2** Monthly proliferative multiple of ginseng hairy roots in C, G and DG groups induced by different concentrations of ginsenosides. Significant compared with C group (a), compared of different concentrations within groups (b), and between G and DG groups under the same concentration (c).
